# Unemployment Insurance

**Published:** 1912-10-19

**Authors:** 


### OUR NATIONAL INSURANCE ACT DEPARTMENT.

### XXXI.—Unemployment Insurance.

Since the commencement of our National Insurance Act Department in March last we have dealt in these articles with topics exclusively connected with the scheme of National Health Insurance, and have ignored the subject of unemployment insurance. We have had good reasons for thus deferring consideration of Part II. of the National Insurance Act, 1911. In the first place, The Hospital is so intimately concerned in all matters relating to public health that we felt it encumbent upon us to examine in detail the provisions of the Act which affect the health and well-being of so large a proportion of the population. There was a second and a very important reason for giving so much attention to Part I. of the Act at the expense of excluding the consideration of Part II., and that was, that so many more of our readers are affected personally by the health insurance provisions than by the unemployment section. We recognised at the outset that it was a matter of paramount importance that our readers should have at their disposal the best advice as to the way in which they could derive the greatest benefit under the Act, and we trust that our efforts in this direction have not been entirely without result. We have at least endeavoured to show that to become a member of an approved society is the only way to derive the full benefit of the insurance, and we have set forward what appears to us to be the guiding principles as to the choice of a society.

The time allowed to those who were employed at the commencement of the Act in which to choose an approved society has now expired, and there is, in consequence, no further need for us to deal with the question.

Those of our readers who come within the scope of the unemployment provisions of the Act did not stand in need of advice of this nature in regard to their insurance against unemployment, for the basis of the administration of Part II. of the Act is the Labour Exchange and not the approved societies.

We had still a further reason for our attitude in ignoring unemployment insurance—namely, the large amount of public attention devoted to the health provisions and the scant consideration accorded in the public press to matters arising out of the operation of Part II. of the Act.

### The Problems of Unemployment.

The question of insurance against unemployment is, however, a matter of considerable social interest, and presents a number of problems of a fascinating character. We propose, therefore, to turn our attention during the next few weeks to this aspect of the National Insurance Act, and in so doing it will be our object not only to explain its provisions, but to indicate the nature of some of the social problems to which we have just referred, and the propriety of meeting their solution by means of insurance.

It will, doubtless, be remembered that the National Insurance Act was criticised for including in the same measure two such dissimilar objects as health and unemployment insurance. This dissimilarity is fundamental, for while all persons are liable to sickness and disease it is not everyone, even amongst the employed classes, who is subject to the risk of unemployment or to such risk, at least, as is capable of insurance. In spite of this fundamental difference there is much that is common to both. Sickness and unemployment act and interact on each other. There is, for instance, no more fruitful cause of sickness than unemployment, for unemployment means loss of wages and of the means of providing necessary bodily nourishment, which, in turn, leads to loss of power to withstand the disintegrating forces of disease. Conversely, the lack of physical vigour, due to ill-health, undoubtedly affects the capacity for work, and the person who is incapable of work is the one who is thrown out of employment. This was apparently the reason for the inclusion in a great scheme for National Health Insurance of a tentative and experimental scheme for dealing with the evils of unemployment.

### The Lack of Public Interest.

We have already alluded to the comparatively overwhelming interest that has been taken in the public press in the health insurance provisions. The reasons for this are not far to seek. Part II. of the Act is sectional in its application, that is to say, insurance against unemployment is made compulsory for the present only so far as those who are engaged as workmen in the specified occupations of building, construction of works, shipbuilding, mechanical engineering, ironfounding, construction of vehicles, and sawmilling. As thus limited, the number of those who will be compulsorily insured is estimated at about 2,500,000, as against an estimate of about 14,000,000 of insured persons coming under the provisions of Part I. of the Act.

Then, again, the detailed discussion of the various sections of the unemployment provisions was not conducted in a Committee of the whole House of Commons, but was relegated to a Standing Committee, whose deliberations were conducted at the same time as those of the House on the more important health insurance, and the air of publicity was therefore wanting. Furthermore, there has been nothing of a sensational character, such as the negotiations between the Chancellor of the Exchequer and the medical profession to stimulate public attention.

It is not to be wondered at, therefore, that there is wide prevailing ignorance of this aspect of the recent legislative enactment, and if our remarks do no more than to enable our readers to take an intelligent interest in this new branch of social legislation they will have served a useful purpose.

There is little doubt that the credit of authorship of Part II. of the National Insurance Act, 1911, is due to Sir H. Llewellyn Smith, K.C.B., of the Board of Trade, for in a masterly presidential address delivered to the Economic Science and Statistics Section of the British Association at Sheffield in 1910 he clearly set forth the fundamental problems that had to be considered and adumbrated the lines on which unemployment insurance was practicable. In all essential matters the Act follows the recommendations then put forward. In attacking the problem the author pointed out that the object of insurance is the increase of security, and he did not seek to justify it as an end in itself, nor beyond the limits of removing from the shoulders of the worker the risks which the latter is unable to prevent, and which, even if foreseen, is incompetent to forestall by making unaided an adequate provision.

Thus he drew a line of distinction between unemployment due to personal incompetence and that arising from widespread depression of trade. "I think," he said, "that most people would agree that the personal risk of losing employment through bad work, irregular attendance, or drunken habits is one which is absolutely necessary in the public interest to leave attached in all its force to the individual workman. On the other hand, I think that most people would agree that in a country like the United Kingdom at the present time the incalculable risk of a prolonged depression of trade, due perhaps to some financial catastrophe thousands of miles away, is one the exposure to which of the individual workman does little but harm." Here, then, is the first criterion of a satisfactory scheme of unemployment insurance; it must not weaken the individual responsibility of the workman, whilst, on the other hand, it should be so framed as to provide added security against risks over which he has no control.

Is Unemployment Insurance Desirable?

Examination shows that it is by no means an easy matter to determine what risks come within the description of desirable "insurance risks." Trade depressions do not affect all branches of industry to the same extent, or, for that matter, in the same direction, and in consequence the necessity for unemployment insurance, in the nature of spreading the surplus earnings of good trade years over periods of depression in such a way as to equalise the wages of the workers, is a matter depending upon the particular trade or industry. Furthermore, it has to be recognised that it is not only a general depression of trade that exercises an influence on the unemployment of individuals. There may be local causes, such, for instance, as the staple industry of a particular locality falling into decay. No scheme of insurance against unemployment arising from this cause could remain economically sound without due provision of safeguards for ensuring the mobility of labour. Again, dealing with the question from the point of view of the individual, there is no doubt that unemployment is to a considerable extent a function of the age of the worker. With increasing age there is greater liability to the risk of unemployment, though it can hardly be said that the difference is sufficient to warrant the graduation of benefits or contributions to an unemployment fund with reference to the age of the worker.

Sir H. Llewellyn Smith concluded his most interesting address in the following summary of the essential characteristics of any unemployment insurance scheme:—

"1. The scheme must be compulsory; otherwise the bad personal risks against which we must always be on our guard would be certain to predominate.

2. The scheme must be contributory, for only by exacting rigorously as a necessary qualification for benefit that a sufficient number of weeks' contributions shall have been paid by each recipient can we possibly hope to put limits on the exceptionally bad risks.

3. With the same object in view there must be a maximum limit to the amount of benefit which can be drawn both absolutely and in relation to the amount of contribution paid . . . this will automatically exclude the loafer.

4. The scheme must avoid encouraging unemployment, and for this purpose it is essential that the rate of unemployment benefit payable shall be relatively low.

5. For the same reason it is essential to enlist the interest of all those engaged in the insured trades, whether as employers or as workmen, in reducing unemployment, by associating them with the scheme both as regards contributions and management.

6. As it appears that some trades are more suitable to be dealt with by insurance than others . . . for the scheme to have the best chance of success, it should be based upon the trade group, and should at the outset be partial in operation.

7. The group of trades to which the scheme is to be applied must, however, be a large one and must extend throughout the United Kingdom, as it is essential that industrial mobility as between occupations and districts should not be unduly checked.

8. A State subvention and guarantee will be necessary . . . in order to give the necessary stability and security. . . .

9. The scheme must aim at encouraging the regular employer and workman and discriminating against casual engagements. . . .

10. The scheme must not act as a discouragement to voluntary provision for unemployment, and for that purpose some well-devised plan of co-operation is essential between the State organisation and the voluntary associations which at present provide unemployment benefit for their members."

It would be hard to devise a clearer statement of the essential principles of unemployment insurance, and we shall see in our next few issues how they have been given practical shape and expression.

